# Reversibility of Antipsychotic-Induced Weight Gain: A Systematic Review and Meta-Analysis

**DOI:** 10.3389/fendo.2021.577919

**Published:** 2021-07-28

**Authors:** Helene Speyer, Casper Westergaard, Nikolai Albert, Mette Karlsen, Anne Emilie Stürup, Merete Nordentoft, Jesper Krogh

**Affiliations:** ^1^Mental Health Centre Copenhagen, Copenhagen Research Center for Mental Health (CORE) Research Unit, Copenhagen University Hospital, Hellerup, Denmark; ^2^Department of First Episode Psychosis, Psychiatric Centre, Glostrup, Denmark; ^3^Department of Endocrinology and Metabolism, Rigshospitalet, University of Copenhagen, Copenhagen, Denmark

**Keywords:** antipsychotic medication, weight loss, obesity, antipsychotic induced weight gain, discontinuation, dose reduction

## Abstract

**Background and Aims:**

Weight gain is a major adverse effect of antipsychotic medication, negatively affecting physical and mental well-being. The objective of this study was to explore if dose reduction, discontinuation, switch to a partial agonist, or switch from polypharmacy to monotherapy will lead to weight loss.

**Methods:**

Controlled and uncontrolled studies reporting the effects of discontinuation, dose reduction, switch to a partial agonist, or switch from polypharmacy to monotherapy on weight were included. Primary outcome was difference in weight compared to maintenance groups based on controlled studies. Secondary outcome was change in weight from initiation of one of the included interventions until follow-up in a pre-post analysis.

**Results:**

We identified 40 randomized controlled trials and 15 uncontrolled studies including 12,279 individuals. The effect of the interventions, *i.e.* dose reduction, drug discontinuation, or switch to a partial agonis, reduced the weight with 1.5 kg (95% CI −2.03 to −0.98; P < 0.001) compared to maintenance treatment. The weight change from pre to post was a reduction of 1.13 kg (95% CI −1.36 to −0.90; P < 0.001).

**Conclusion:**

We found a significant but small reduction in weight, suggesting that antipsychotic-induced weight gain can be reversed to some degree. Only a few studies were designed to address the question as primary outcome, which limits the generalizability of our findings.

## Introduction

Shared decision making is the cornerstone of modern, evidence-based medicine and requires transparency about risks and benefits associated with any treatment decisions. Second generation antipsychotic medication is the mainstay of treatment of psychotic disorders (1) and is increasingly prescribed for other indications like bipolar disorder and severe depression and off-label indications such as sleeplessness and anxiety (2, 3). Weight gain is a major adverse effect of second-generation antipsychotics, affecting quality of life (4), personal recovery, and somatic morbidity and is a common reason for antipsychotic discontinuation (5). Therefore, knowledge about the reversibility of antipsychotic weight gain is important to qualify the informed decision of both initiation and discontinuation.

Shared decision-making refers to a process where expert knowledge, data from the literature, and the patient’s personal values and preferences are integrated in a final decision. Information about the risk of weight gain during treatment is important, and it is well documented in the scientific literature (6). However, knowledge about the reversibility of weight gain when the antipsychotic medication is reduced or discontinued remains scarce. To fully inform decisions to start or to stop medication, information about reversibility is crucial. Many may expect adverse effects to be reversible upon discontinuation, but this is not always the case with antipsychotic medication, as exemplified by tardive dyskinesia (7). Non-pharmacological interventions to reduce weight in a general population have shown only transient effect on obesity (8). While decades have passed with different trials of behavioral interventions, it is increasingly realized that temporary lost weight is normally rapidly regained, and obesity is now defined as: “chronic, relapsing, multi-factorial, neurobehavioral disease, wherein an increase in body fat promotes adipose tissue dysfunction and abnormal fat mass physical forces, resulting in adverse metabolic, biomechanical, and psychosocial health consequences.” (https://obesitymedicine.org/obesity-algorithm/).

If antipsychotic-induced obesity is an irreversible adverse effect, it has consequences for the shared decision making and potentially also for the off-label prescribing. If there is a risk of developing life long, severe obesity, some might find the risk–benefit balance to tip against medication. On the other hand, if the patient considers discontinuing antipsychotic medication to lose weight, it is important to know if this is likely to happen, to balance against the risk of relapse.

None of the second-generation antipsychotics are weight neutral, but some are more obesogenic than others, with olanzapine and clozapine having the highest obesogenic properties. Substantial effort has been put into understanding the molecular mechanisms underlying the obesogenic properties of antipsychotic medication, without clear answers (9). While the dopamine blockade produces the antipsychotic effect, several other neurotransmitters like the muscarinic, serotonergic, and histaminergic systems account for the metabolic effects, but no definite explanations have been established (10). Recently, intense focus has been put on the gut microbiota, which is linked to antipsychotic medication, obesity, and even to clinical variables of schizophrenia (11), suggesting that the effect of antipsychotic drugs is mediated *via* an effect on the gut microbiome (12) and then affecting psychotic symptoms and obesity.

Peculiarly, little research has been focusing on the potential reversibility of weight gain if the antipsychotic drug is reduced, discontinued, or switched to an agent with lower obesogenic properties, but case reports (13, 14) and a few studies of patients with intellectual disabilities and children with bipolar disorder (15) suggest that at least some weight can be lost after discontinuation.

### How the Interventions May Work

Reducing or discontinuing second generation antipsychotic medication may cause weight loss due to negative energy balance or directly through affecting various neurotransmitters and neuroendocrine signaling. Negative energy balance could be achieved by decreased appetite and by increased physical activity due to lower level of sedation. The effect is likely mediated *via* neurotransmitter signaling involving histamine and serotonin (16), and some suggest that thyroid function is associated with antipsychotic medication (17). Similarly, switch to partial agonists may affect weight through lower affinity to histamine receptors and lower level of sedation.

### Objective

The aim of this systematic review was to report the effect of dose reduction/discontinuation of second-generation antipsychotic, switch to a partial dopamine agonist, or switch from polypharmacy to monotherapy on weight in individuals diagnosed with severe mental illness. We hypothesized that any of the four interventions would lead to a reduction in weight, in relative values when compared to maintenance treatment and in absolute values when compared to weight before interventions.

## Method

### Eligibility

#### Inclusion Criteria

1. Patients diagnosed with major depression, schizophrenia, psychosis, bipolar disorder or schizoaffective psychosis, aged above 17 years old.
2. Interventions evaluating the effect of the following interventions were included:
  - Dose reduction, defined as interventions where dose was gradually reduced until completely stopped, or a smaller dose was kept due to re-emergence of symptoms.
  - Switch from antipsychotic polypharmacy to monotherapy, defined as any intervention where all participants were abruptly or gradually switched from two antipsychotic medications at any dose, where at least one was a second-generation antipsychotic to one antipsychotic drug.
  - Discontinuation, defined as interventions where all participants where gradually or abruptly discontinued.
  - Switch from any second generation antipsychotic to a partial dopamine agonist: aripiprazole, brexpiprazole, or cariprazine (18).
3. A minimum of four weeks exposure to a second-generation antipsychotic prior to discontinuation.
4. Control condition was defined as maintenance treatment with antipsychotic medication.
5. Outcomes for weight should be reported in kg or lbs. as endpoint or change scores.
6. Clinical studies on humans, including cohort studies, non-randomized controlled trials, and randomized controlled trials reporting either between groups at end point or pre- post change scores.

#### Exclusion Criteria

1. Interventions evaluating the effect of intermittent treatment.
2. Studies where only clinically significant weight change was reported as dichotomous outcome.

### Information Sources

The bibliographical search was performed on 18^th^ of February 2021 and included a search of PUBMED, Scopus, Lilac, Embase, and Web of Science using medical subject headings (MESH or similar) when possible and text word terms*: (Major depression OR schizophrenia OR bipolar Or schizoaffective OR psychosis OR severe mental disorder) AND (deprescription OR deprescribing OR discontinuation OR dose reduction OR cessation OR reduction OR switching OR tapering OR polypharmacy OR dose reduction OR reducing medication OR ceasing medication OR switch to monotherapy OR monotherapy OR withdrawal OR switch to aripiprazole OR relapse prevention OR maintenance) AND (second generation antipsychotic OR antipsychotic OR neuroleptic OR olanzapine OR clozapine OR aripiprazole OR amisulpride OR zotepine OR second generation antipsychotic OR quetiapine OR paliperidone OR lurasidone OR risperidone OR ziprasidone OR sertindole OR brexpiprazole OR cariprazine OR asenapine OR iloperidone)*.

### Study Selection

Two authors (HS, CW) independently examined the remaining full list, selected relevant abstracts and examined the relevant full text determining compliance with inclusion criteria using the software from Covidence.

### Data Extraction

Two authors (HS, CW) independently extracted data using Covidence software. The authors were not blinded to study results, authors, or institutions. Data extraction included data on weight and additional information regarding age, gender, number of patients, duration of treatment, type of treatment, baseline weight, type of discontinuation and duration of follow up. HS and CW independently conducted risk of bias applying Rob-2 tool (19).

### Outcomes

Primary outcome was defined as mean difference in weight between any of the methods of: (discontinuation, dose reduction, switch to partial agonist, switch to monotherapy) as described in the *Introduction*, compared to maintenance treatment measured on a continuous scale in kg. Results for each of the four interventions will be reported both pooled and separately. Secondary outcome was pre-post change in weight from initiation of intervention to end of follow-up measured on a continuous scale in kg reported pooled and separately for each of the four groups. This analysis included non-controlled intervention studies pooled with the intervention groups from the controlled trials. Type of intervention, diagnoses and difference in duration of exposure in active *versus* placebo medication was tested as potential moderators of effect.

### Data Synthesis

In order to include a maximum of studies, we combined end-scores and change scores for primary outcomes. Thereby, we abstain from calculating standardized measures, as the combination of dispersion of end- and change scores cannot be combined in a standardized effect size (20). If change scores as well as end scores were reported, end-scores were preferred. All results were reported with 95% confidence intervals and 95% prediction intervals (21). Missing measures of dispersion were imputed as recommended by Cochrane (20). Random effects were reported, assuming underlying heterogeneity of effects due to variations in the interventions. The degree of heterogeneity was quantified using the I^2^ statistic, which can be interpreted as the percentage of variation observed between the trials attributable to between-trial differences, rather than sampling error (chance). Heterogeneity was explored by analyses of subgroups and meta-regressions. Results from randomized clinical trials and uncontrolled studies were analyzed and reported separately. Results from RCTs were pooled with pre-post studies if data for these were available.

Data was analyzed using Comprehensive Meta-Analysis v. 3.11; p-values <0.05 were considered significant. Prisma reporting guidelines were followed (22).

### Deviations From the Protocol

1) We changed the switch to partial agonist from only including aripiprazole to also including brexpiprazole and cariprazine. 2) We changed one moderator from study duration to difference in exposure to drug in active *versus* placebo drug. 3) An improved search strategy was applied.

## Results

**Figure 1:**
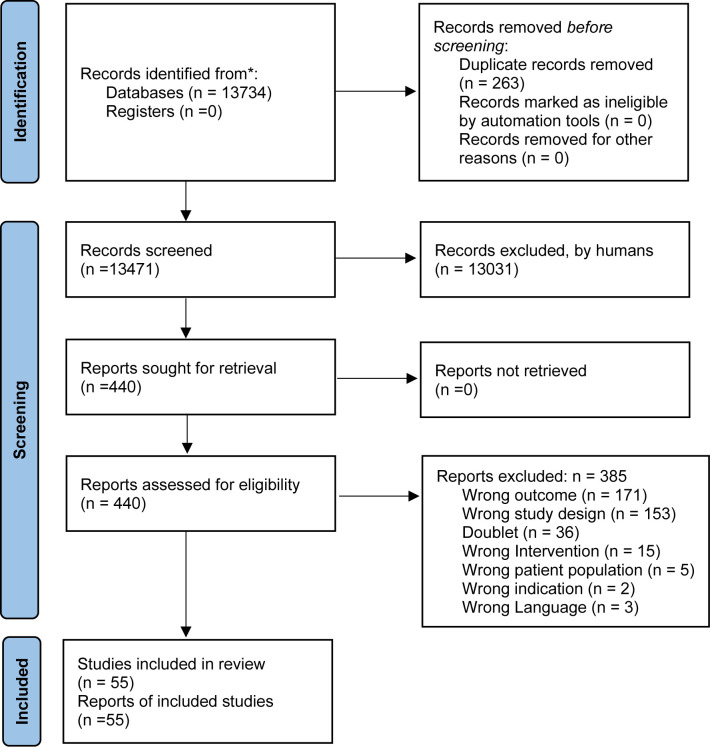
Flowchart of literature search and study selection.

The main bibliographical search was conducted on 18^th^ February 2021. As illustrated in **Figure 1**, we identified 55 studies (23–76) fulfilling the inclusion criteria and none of the exclusion criteria (**Table 1**). Of these, 40 were RCTs and 15 were uncontrolled studies. Of these, 33 reported on the effect of discontinuation, seven on dose reduction, 18 on switch to a partial agonist, and no studies on switch from polypharmacy to monotherapy. A total of 12,279 (mean participants 224 per study) participants were included, with a mean age of 40 years old, and the mean baseline weight of 69.6 kg.

**Table 1:** Characteristics of included studies.

Author	Participants	Baseline weight	Type of intervention	Duration of follow-up (week)	Antipsychotic	Design	N at baseline
Alexopoulos et al. (51)	Major depression	NA	Drug *versus* placebo in maintenance phase	24	Risperidone	Randomized	63
	Mean age 63.4						
	44% male						
Beasley et al. (23)	Schizophrenia/schizoaffective	NA	Drug *versus* placebo in maintenance phase	52	Olanzapine	Randomized	326
	Mean age 35.7						
	53% male						
Berwaerst et al. (36)	Bipolar	78 kg	Drug *versus* placebo in maintenance phase	170	Risperidone	Randomized	300
	Mean age 40						
	45% male						
Brunner et al. (41)	Major depression	83.1 kg	Drug *versus* placebo in maintenance phase	27	Olanzapine	Randomized	444
	Mean age 44						
	33.1% male						
Calabrese et al. (37)	Bipolar	89.2	Drug *versus* placebo in maintenance phase	52	Aripiprazole	Randomized	266
	Mean age 40.6						
	42.5% male						
Carlson et al. (63)	Bipolar	86.6	Drug *versus* placebo in maintenance phase	52	Aripiprazole	Randomized	351
	Mean age 39						
	35% male						
Chen et al. (24)	Schizophrenia	66.0	Dose reduction *versus* maintenance	52	Quetiapine	Randomized	178
	Mean age 24.2						
	45% male						
Chen et al. (69)	Schizophrenia/bipolar	98.5	Switch to aripiprazole	52	Second generation antipsychotic	No control group	24
	Mean age 45.3						
	45,8% male						
Casey et al. (26)	Schizophrenia	90.2	Switch to aripiprazole	8		Randomized	311
	Mean age 39						
	70% male						
Cooper et al. (75)	Schizophrenia	76.7	Drug *versus* placebo in maintenance phase	26	Zotepine	Randomized	121
	mean age 42.3						
	68% male						
Durgham et al. (53)	Schizophrenia	75.3	Drug *versus* placebo in maintenance phase	72	Cariprazine	Randomized	200
	Mean age 38						
	66% male						
Fleischhacker et al. (55)	Schizophrenia	82.9	Drug *versus* placebo in maintenance phase	52	Brexpiprazole	Randomized	202
	Mean age 39.7						
	61% male						
Flint et al. (32)	Major depression	77.3	Drug *versus* placebo in maintenance phase	36	Olanzapine	Randomized	126
	Mean age 55.3						
	38.1% male						
Fu et al. (46)	Schizoaffective	78.6	Drug *versus* placebo in maintenance phase	60	Paliperidone	Randomized	334
	Mean age 38.6						
	50.6% male						
Ganguli et al. (76)	Schizophrenia	47.8	Switch to aripiprazole	8		No control group	33
	NA						
	28% male						
Graff-Guerrero et al. (59)	Schizophrenia	83.8	Dose reduction	Variable	Olanzapine/Risperidone	No control group	35
	Mean age 60						
	74% male						
Hwang et al. (49)	Schizophrenia	66 kg	Switch to aripiprazole	8	Aripiprazole	No control group	79
	Mean age 39						
	40.6% male						
Ishigooka et al. (66)	Schizophrenia	65.5	Switch to brexpiprazole	56	Brexpiprazole	No control group	47
	Mean age 49						
	51% male						
Kane et al. (43)	Schizophrenia	77,7 kg	Drug *versus* placebo in maintenance phase	24 weeks	Olanzapine	Randomized	466
	Mean age 39 years						
	66% male						
Kane et al. (44)	Schizophrenia	76,5	Drug *versus* placebo in maintenance phase	26	Asenapine	Randomized	386
	Mean age 39						
	57.4% male						
Kane et al. (57)	Schizophrenia	83,8	Drug *versus* placebo in maintenance phase	52	Aripiprazole	Randomized	403
	Mean age 40.9						
	59.6% male						
Keck et al. (50)	Bipolar	85.7 kg	Drug *versus* placebo in maintenance phase	74	Aripiprazole	Randomized	151
	Mean age 39.6						
	33% male						
Kim et al. (28)	Schizophrenia	104.1	Switch to aripiprazole	16	Aripiprazole	No control group	15
	Mean age 34						
	60% male						
Kim et al. (30)	Schizophrenia	67 kg	Switch to aripiprazole	26		No control group	61
	Mean age 30.8						
	44.3% male						
Kramer et al. (42)	Schizophrenia	79,5	Drug *versus* placebo in maintenance phase	52	Paliperidone	Randomized	113
	Mean age 38.2						
	59% male						
Liebowitz et al. (71)	Major depression	83 kg	Drug *versus* placebo in maintenance phase	52	Quetiapine	Randomized	776
	Mean age 44.6 years						
	34% male						
Macfadden et al. (38)	Bipolar	70	Drug *versus* placebo in maintenance phase	52	Risperidone	Randomized	124
	Mean age 38.7						
	71.8% male						
Marcus et al. (70)	Bipolar	81	Drug *versus* placebo in maintenance phase	52	Aripiprazole	Randomized	337
	39 years						
	45.1% male						
Newcomer et al. (72)	Schizophrenia/schizoaffective	92	Switch to aripiprazole	16	Olanzapine	Randomized	173
	Mean age 39.2						
	64.2% male						
Ozawa et al. (73)	Schizophrenia	57	Dose reduction *versus* maintenance	52	Olanzapine/Risperidone	Randomized	35
	Mean age 64						
	66% male						
Pae et al. (29)	Schizophrenia	66.4 kg	Switch to aripiprazole	12		Randomized	77
	Mean age 36.4						
	42,8% male						
Peuskens et al. (54)	Schizophrenia	70	Drug *versus* placebo in maintenance phase	24	Quetiapine	Randomized	197
	Mean age 36						
	60% male						
Pigott et al. (35)	Schizophrenia	75	Drug *versus* placebo in maintenance phase	26	Aripiprazole	Randomized	310
	Mean age 42 years						
	56.1% male						
Quiroz et al. (33)	Bipolar	75.1 kg	Drug *versus* placebo in maintenance phase	96	Risperidone	Randomized	303
	Mean age 39						
	51% male						
Rapaport et al. (45)	Major depression	NA	Drug *versus* placebo in maintenance phase	24	Risperidone	Randomized	243
	Mean age 48.3						
	36,2% male						
Rouillon et al. (56)	Schizophrenia	76.4	Dose reduction *versus* maintenance	24	Olanzapine	Randomized	97
	Mean age 39.3						
	68.3% male						
Ryckman et al. (67)	Schizophrenia	80.3 kg	Switch to aripiprazole	12	Aripiprazole	No control group	400
	Mean age 41						
	56% male						
Rui et al. (68)	Schizophrenia	63.4	Drug *versus* placebo in maintenance phase	52	Paliperidone	Randomized	136
	Mean age 31						
	41% male						
Schorr et al. (61)	Schizophrenia	97.0	Switch to aripiprazole	12		No control group	53
	Mean age 35						
	60% male						
Spurling et al. (27)	Schizophrenia/bipolar	NA	Switch to aripiprazole	24		No control group	23
	Mean age 45						
	67% male						
Stroup et al. (77)	Schizophrenia/schizoaffective	103	Switch to aripiprazole	24	Olanzapine/Risperidone/Quetiapine	Randomized	205
	Mean age 41						
	63.7% male						
Suppes et al. (64)	Bipolar	86.4	Drug *versus* placebo in maintenance phase	104	Quetiapine	Randomized	628
	Mean age 40.1						
	47.5% male						
Suzuki et al. (60)	Schizophrenia	62.5	Switch to aripiprazole	Variable		No control group	19
	NA						
	NA						
Takeuchi (34)	Schizophrenia	68.8	Dose reduction *versus* maintenance	28	Olanzapine/Risperidone	Randomized	61
	Mean age 39						
	60% male						
Takeuchi et al. (31)	Schizophrenia	63.1	Switch to aripiprazole	52		No control group	32
	Mean age 27						
	56.2% male						
Tohen et al. (39)	Bipolar	NA	Drug *versus* placebo in maintenance phase	72	Olanzapine	Randomized	99
	Mean age 41.2						
	48.3% male						
Tohen et al. (48)	Bipolar		Drug *versus* placebo in maintenance phase	48	Olanzapine	Randomized	356
	Mean age 40.4						
	39% male						
Tsuboi et al. (47)	Schizophrenia	60.2 kg	Dose reduction *versus* maintenance	52	Olanzapine/Risperidone	Randomized	68
	Mean age 55.4						
	60.3% male						
Wang et al. (58)	Schizophrenia		Dose reduction *versus* maintenance	52	Risperidone	Randomized	404
	Mean age 32.6	NA					
	46% male						
Woo et al. (74)	Bipolar	70.9	Switch to aripiprazole	24	Aripiprazole	No control group	77
	Mean age 36						
	38% male						
Weisler et al. (25)	Bipolar	71.6	Drug *versus* placebo in maintenance phase	104	Quetiapine	No control group	808
	Mean age 39.5						
	46.7% male						
Weiden et al. (40)	Schizophrenia	75.4 kg	Drug *versus* placebo in maintenance phase	24	Iloperidone	Randomized	303
	Mean age 38,3						
	58.7% male						
Weiss et al. (65)	Schizophrenia	Na	Drug *versus* placebo in maintenance phase	24	Brexpiprazole	Randomized	202
	NA						
	NA						
Young et al. (52)	Bipolar	78.8	Drug *versus* placebo in maintenance phase	52	Quetiapine	Randomized	584
	Mean age 40.3						
	41.2% male						
Yatham et al. (62)	Bipolar	80.3	Drug *versus* placebo in maintenance phase	52	Aripiprazole	Randomized	105
	Mean age 37.9						
	50% male						

### Risk of Bias

The risk of bias assessment is presented in **Table S1** (19). We found a low risk of bias in 26 trials, and some concerns in 14 trials. All the included trials were blinded, but as weight was reported as adverse events, the methods to handle missing data were often not clearly described. Visual inspection of Funnel plots (**Figures S1** and **S2**) did not reveal any signs of publication bias.

### Primary and Secondary Outcomes

**Figure 2:**
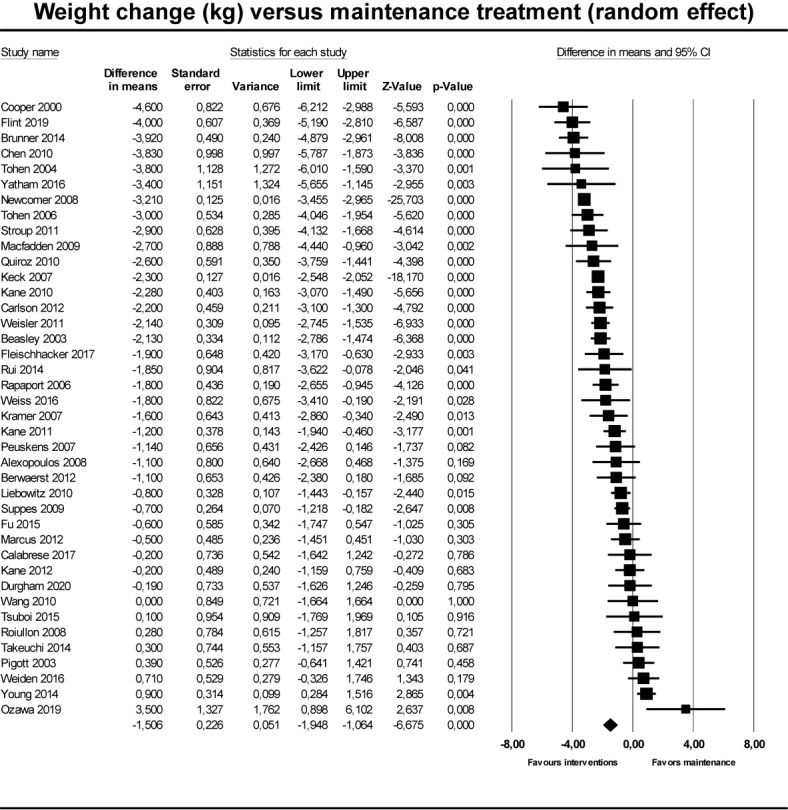
Forest plot showing results from studies comparing maintenaince treatment to dose reduction, switch or discontinuation.

For the primary outcome (**Figure 2**), weight change in intervention groups compared to maintenance treatment, based on 40 randomized trials, we found a weight reduction of -1.51 kg (95% CI -1.95 to -1.06) in groups who had their medication discontinued/reduced/switched to partial agonist, compared to control groups who continued maintenance treatment (**Table 2**). The corresponding 95% prediction interval was −4.71 to 1.61. In the subgroup analyses, the effect for discontinuation (N = 31) was −1.60 kg (95% CI −2.25 to −0.95; P < 0.001), for dose reduction (N = 7) −0.46 kg (95% CI −2.0 to 1.07; P = 0.56), and for switch to partial agonist (N = 2) it was −3.19 kg (95% CI −3.43 to −2.96; P < 0.001).

**Table 2:** Pooled results of primary outcomes, pooled and grouped by type of intervention) measured in kilograms (kg) with standard error (SE).

	No. of studies	Point estimate (kg) *versus* maintenance	SE	95% CI	*p*	I^2^ (%)
Pooled effect	40	−1.51	0.23	−1.95 to −1.06	P < 0.00	90.7
Discontinuation	31	−1.57	0.23	−2.02 to −0.98	P < 0.001	
Dose reduction	7	−0.46	0.79	−2.00 to 1.07	P = 0.55	
Switch to partial agonist	2	−3.19	0.12	−3.43 to −2.96	P < 0.001	

**Figure 3:**
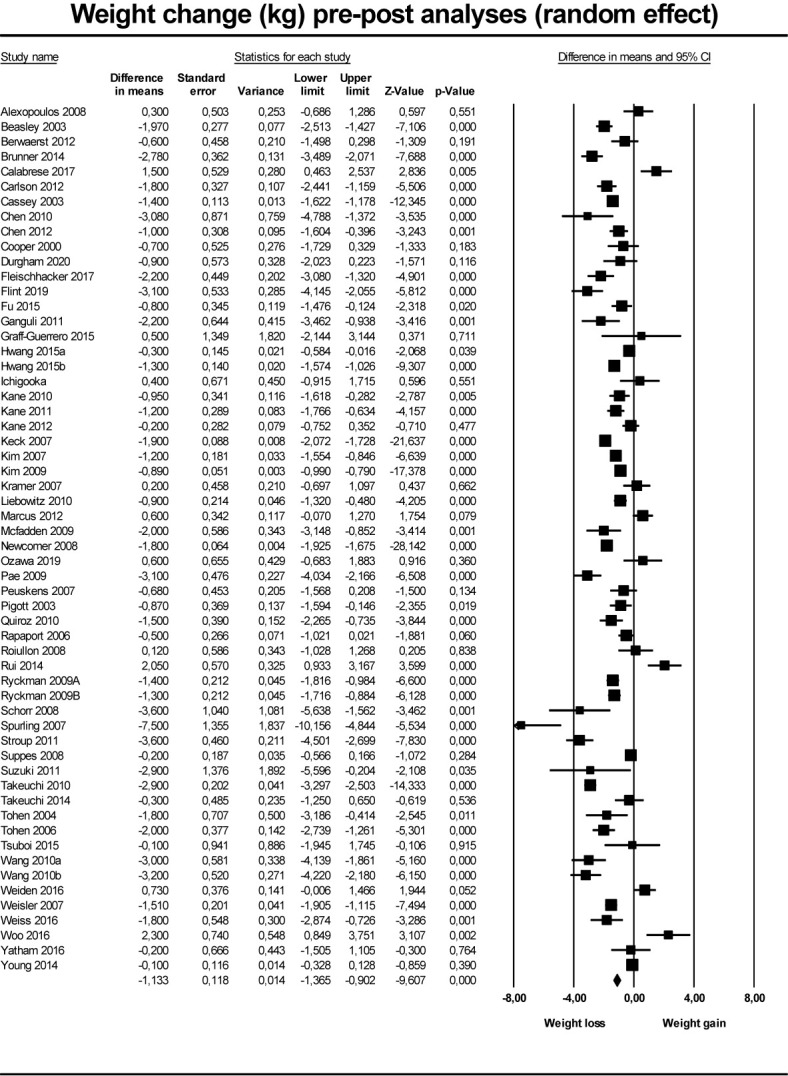
Forest plot showing results from studies comparing pre- to post intervention change in weight.

For the secondary outcome (**Figure 3**), pre-post analyses based on 55 studies reporting on 58 groups (three studies had two intervention groups), the pooled results of interventions including discontinuation, dose reduction, and switch to partial agonist, found a weight reduction of 1.13 kg (95% CI −1.36 to −0.90; P < 0.001) at end of follow-up compared to baseline values (**Table 3**). In the subgroup analyses, the effect of discontinuation (N = 33) was −0.86 kg (95% CI −1.34 to −0.38; P < 0.001), of dose reduction (N = 7) was −1.25 kg (95% CI −2.66 to 0.17; P = 0.084), and of switch to partial agonist (N = 18) was −.57 kg (95% CI −1.46 to −0.83; P<0.001).

**Table 3:** Pooled results of secondary outcomes, pooled and grouped by type of intervention) measured in kilograms (kg) with standard error (SE).

	No. of Studies	Point estimates (kg) pre-post	SE	95% CI	*p*	I^2^ (%)
Pooled effect	58	−1.13	0.12	−1.36 to −0.90	P < 0.001	92.8
Discontinuation	33	−0.86	0.18	−1.34 to −0.38	P < 0.001	
Dose reduction	7	−1.25	0.72	−2.66 to 0.17	P = 0.08	
Switch to partial agonist	18	−1.57	0.19	−1.94 to −1.20	P < 0.001	

95% Confidence intervals (95% CIs) and measure of heterogeneity (I^2^).

### Heterogeneity, Subgroup Analyses, and Meta-Regression

The I^2^ was 90.7% for the primary outcome and 97.8% for the secondary outcome, suggesting substantial heterogeneity. This was expected, as we pooled different diagnoses, different interventions, and different designs. We explored heterogeneity by testing categorical variables (diagnoses, controlled *vs*. uncontrolled studies) and one continuous variable (differences in exposure between active and placebo), **Table 4**.

**Table 4:** Results for subgroup analyses and meta-regressions, including weight in kilogram (kg) grouped by diagnoses,and type of study design, standard error (SE), 95% confidence intervals (95% CI).

	No. of Studies	Point estimates (kg)	SE	95% CI	*p*
**Weight (kg) compared to maintenance**					
Schizophrenia	22	−1.16	0.36	−1.87 to −0.46	p = 0.001
Bipolar	13	−1.70	0.38	−2.43 to 0.96	p < 0.001
Depression	5	−2.35	0.84	−3.98 to −0.71	p = 0.005
**Weight (kg) pre-post analysis**					
Controlled trials	41	−1.03	0.19	−1.41 to −0.65	p < 0.001
Uncontrolled trials	17	−1.39	0.20	−1.77 to −1.00	p < 0.001

95% Confidence intervals (95% CIs) and measure of heterogeneity (I^2^).

For the pooled primary outcome, comparing groups based on diagnoses revealed similar effects where studies on schizophrenia (N = 22) had a weight reduction of −2.24 kg (95% CI −0.46 to −3.26; p = 0.001) on bipolar (N = 13) a weight reduction of −1.70 kg (95% CI −2.43 to −0.896 p < 0.001) and on major depression (N = 5) a weight reduction of −2.35 kg (95% CI −3.98 to −0.71; p = 0.005). The differences in exposure between active drug and placebo in the RCTs (N = 17) explained 9% of variation, but this was not significant (p = 0.27).

For the secondary outcome, there was no difference in effect when comparing groups from randomized trials to uncontrolled studies; −1.03 kg *versus* −1.39 kg (p = 0.20).

## Discussion

We found that the pooled effect of discontinuation, dose reduction, and switch to partial agonists reduced the weight with 1.54 kg compared to maintenance treatments with a second generation antipsychotic drug. The 95% CI interval was −2.08 to −1.2, allowing us to reject the null hypothesis, but the corresponding 95% prediction interval was −4.71 to 1.61, which does not exclude the probability of null effect in future trials, but the results could be as high as −4.7 kg. The subgroup results suggest that the effect of switch to partial agonists was larger than discontinuation and dose reduction, but this may reflect trial methodology rather than true differences, as discussed beneath. In the pooled pre-post analysis, we found a weight reduction of 1.13 (p < 0.001), with no substantial differences in subgroups.

The primary outcome, based on controlled trials, and secondary outcomes, based on uncontrolled trials, address two fundamentally different questions with different inherent methodological limitations to the answers. The primary outcome asks the clinical question: Will my weight be lower if I choose a dose reduction/discontinuation strategy, compared to if I continue the same dose? But the outcome does not inform about a weight loss compared to baseline, as the observed difference may be explained by a larger weight increase in the maintenance group. On the other hand, the secondary outcome, the change in weight form baseline to post intervention, addresses the question: Will I lose weight over time if I reduce dose/discontinue medication?

Our results suggest that a weight loss does occur, but substantial methodological considerations limit the validity of our findings. A major limitation is that most of the trials included in the primary analysis were designed to evaluate relapse prevention of antipsychotic drugs in individuals who received treatment during a short stabilization phase and were defined as responders. It is likely that individuals with severe weight gain were excluded in the stabilization phase due to adverse effects, which could explain the low mean baseline weight, which again could affect the generalizability to a real-world population. It could be argued that the short duration of placebo-treatment is insufficient to evaluate weight loss, as many patients relapse and thus have withdrawn from the study. On the other hand, high degrees of relapses are observed in real world data, and these findings therefore reflect the effectiveness rather than efficacy, as complete adherence is often not realistic. Next, the duration of exposure to study drug, active or placebo, differed due to earlier relapse in placebo groups, leaving shorter time to weight change in placebo *versus* active treatment. Data on duration of exposure was available for 17 of the included trials and did not explain a significant proportion of the variance, although this could be a type II error. Weight change was reported as an adverse event for the large majority of RCTs, and it was generally difficult to assess how missing data were handled, and if the way of reporting weight was pre-registered to avoid any multiple possible methods (continuous, dichotomized, BMI, 7% change) could be affected by financial conflicts of interest, as many of the RCTs were industry sponsored. This possible bias is likely to deflate effect size, as most sponsors would be interested in reporting a lower weight gain in the maintenance groups. Analyses of publication bias did not confirm any concerns but cannot exclude selective reporting. Finally, the generalizability of the population could be affected by the short duration of exposure in the open label, stabilization phase prior to randomization, as duration of prior exposure might be important, as pointed out by Kim et al. (30).

This allows only a short period of weight gain, which could deflate effect sizes compared to real-world populations. On the other hand, it may be easier to lose weight if the time being overweight is shorter, which could oppositely inflate effect size.

By comparing the results with the pre-post analysis, it is tempting to conclude that a weight loss is occurring, and that the observed effect cannot be ascribed to weight gain in the maintenance group. However, important caveats in inferring from pre-post studies should be kept in mind. Results could overestimate the effect due to regression to the mean, skewed loss to follow-up; it could also be confounded by lifestyle or other types of medication. Many of the studies reporting on the effect of switching to partial agonists were uncontrolled with metabolic disturbances being the primary outcome, which increases the risk that the participants engaged in parallel weight reducing behaviors, seen as a manualized co-treatment in one study (77), which could inflate the effect.

We did not identify any studies evaluating the effect of switching from antipsychotic polypharmacy to monotherapy on weight. This is surprising, as there has been increased focus on the lack of evidence supporting superior effect of polypharmacy compared to monopharmacy, and therefore safety in switching (78).

Even though the weight reduction is highly statistically significant, it should be discussed if this weight reduction is clinically important. For the subset of individuals who gain tens of kilograms, the moderate reduction is likely to be ignorable. On the other hand, the magnitude is similar to the magnitude of weight lost from lifestyle interventions (79, 80) and by adding topiramate or metformin to current antipsychotic medication, all being recommended in clinical guidelines (81). Furthermore, the observed effects should be interpreted considering the short duration of most of the trials, with some individuals only receiving placebo treatment for a few weeks before showing signs of impending relapse and subsequently withdrew from the study. Thus, it cannot be excluded that the weight loss could continue over time. The largest weight loss was achieved by switching to partial agonists, which could be explained by longer duration of exposure to antipsychotic medication, making risk of relapse in this group lower compared to trial where an active treatment is compared to placebo.

None of the included studies were designed directly to address the reversibility of antipsychotic-induced weight gain, but some studies have been published in other populations, not included in this paper: Upadhyay et al. (15) followed a large sample of children and adolescents (N = 537) with bipolar disorder who had experienced weight gain while treated with psychotropic medication. At 12 months follow-up after discontinuation of psychotropic medication, weight loss was stabilized, but never returned to baseline, and the authors conclude that it is likely that those who gained significant weight during treatment will stay overweight or obese. Significant improvement in weight was found by De Kuijpers et al. (82) in individuals with intellectual disabilities, where a reduction of dose was associated with weight loss. An improvement in metabolic factors and with no psychiatric deterioration, was reported by Hulvershorn et al. (83) who evaluated the effect of antipsychotic discontinuation in youth with disruptive behavior. However, the majority of these were prescribed medication to treat ADHD, which, in itself will induce weight loss. Finally, case reports describing severe cases of rapid weight gain and metabolic disturbances confirm reversibility, at least to some degree (14, 84).

### Limitations

Adding to the limitations of using primarily relapse prevention studies, there are important limitations in the conduction of this meta-analysis that should be kept in mind. First, we did not pre-register the protocol at Prospero. The protocol was circulated in the author group, and all agreed on the design before the literature search was started. Second, we *a-priori* decided to measure weight change as continuous variable, as dichotomizing continuous variable may inflate effect sizes and decrease power (85). When extracting data, we were surprised to identify a large number of papers only reporting >7% weight gain and not supplementing with 7% weight loss or a mean weight change. It is possible that excluding these papers has introduced a selection bias. Third, we did not assess potential adverse events associated with dose reduction, switch, or discontinuation. To fully inform a clinical decision, potential risks, such as relapses or worsening of substance abuse, are just as important. However, as we chose only to include studies where weight was reported, reporting risks of adverse events on this basis would not provide the full picture of the available literature that has been done by others (86–89). A general finding was that the relative risk of relapse was doubled after discontinuation [27% *vs* placebo 64%; risk ratio (RR) 0.40, 95% CI 0·33–0·49] (90) on short term. However, it has been argued that relapse rates converge with time, and the chance of recovery is increased after dose reduction. Based on very few trials, switch to monotherapy does not seem to increase relapse rates (78, 91). Finally, the lack of access to individual data precludes the possibility of performing subgroup analyses based on personal characteristics like magnitude of weight gain during treatment or type of antipsychotic medication. Thus, it cannot be excluded that individuals with severe gain also have an equivalent weight loss, but that these are hidden in the mean.

### Implications for Research

The lack of direct studies on the reversibility of weight gain calls for future research directly focusing on this topic in rigorously designed studies. Most important, the subgroups with substantial weight gain, for whom this issue may be especially important, need to be included. Ideally, RCTs should be conducted, but the feasibility in conducting these is challenging (92), and observational designs could be an alternative (93). Close monitoring of clinical characteristics during treatment in early intervention services could provide valuable information if interpreted correctly. In order to address, specifically, the question of causality, careful considerations should be put on the potential confounders, and we suggest that transparent frameworks, such as directed acyclic graphs (DAGs) (94), could be applied to make causal inferences based on observational data.

### Implications for Clinical Practice

Any health care decisions are based on information about potential risks and benefits. As weight gain and potential loss are important to many people, it should be shared that little is known about the reversibility of antipsychotic-induced weight gain. This lack of knowledge could affect the decision, especially when drugs are prescribed as off-label (3), where the potential benefits are less clear. Some may worry that patients with severe mental illness may reject the medication in fear of substantial and irreversible weight gain, and thereby put themselves at risk of relapse. However, as long as we regard the patients as having decision capacity (95) and therefore able to provide informed consent, it is our obligation to inform them about potential adverse effects, including what we currently do not know, if this could affect the decision making. In four RCTs determining the effect of dose reduction, a paradoxical weight increase was seen in the reduced group (47, 56, 73, 96), suggesting that metabolic adverse effects may not be dose dependent, which is also found in other studies (97). This underlines the importance of reconsidering the current off-label prescribing trend, as also small doses may lead to metabolic disturbances.

## Conclusion

The main finding of this study is a paucity of studies designed to directly evaluate the reversibility of antipsychotic-induced weight gain. This lack of knowledge is problematic, as individuals making an informed decision has the right to know if they run a risk of lifelong obesity. In spite of the limitations discussed above, our findings do suggest that at least some weight gain is reversible, even though there is doubtfully any health benefit in this small effect size when balanced against the risk of relapse. The lack of dose-related effect is important and should lead to increased awareness of off-label use of low dose second generation antipsychotic for indications like sleep and anxiety, as this might create equally serious weight problems and is increasingly being prescribed (98).

## Data Availability Statement

The original contributions presented in the study are included in the article/**Supplementary Material**, further inquiries can be directed to the corresponding author.

## Author Contributions

HS conceived the idea and made the analysis plan. HS and CW performed the bibliographic search, extracted data, performed the final analyses and drafted the manuscript. All authors contributed to the article and approved the submitted version.

## Conflict of Interest

The authors declare that the research was conducted in the absence of any commercial or financial relationships that could be construed as a potential conflict of interest.

## Publisher’s Note

All claims expressed in this article are solely those of the authors and do not necessarily represent those of their affiliated organizations, or those of the publisher, the editors and the reviewers. Any product that may be evaluated in this article, or claim that may be made by its manufacturer, is not guaranteed or endorsed by the publisher.
