# Supplementary material for: Reversibility of Antipsychotic-Induced Weight Gain: A Systematic Review and Meta-Analysis
Source: Front Endocrinol (Lausanne). 2021 Jul 28;12:577919. doi: 10.3389/fendo.2021.577919 (PMC8355990; doi:10.3389/fendo.2021.577919)
Supplement: Supplementary file 1 [file DataSheet_1.pdf]

| Study ID | D1 | D3 | Overall |
|---|---|---|---|
| Alexopoulos 2008 | + | + | + |
| Beasley 2003 | ! | + | ! |
| Berwaerts 2012 | + | + | + |
| Brunner 2014 | ! | + | ! |
| Calabrese 2017 | + | + | + |
| Chen 2010 | + | + | + |
| Cooper 2000 | + | + | + |
| Durgam 2020 | + | + | + |
| Fleischacker 2017 | + | + | + |
| Flint 2019 | + | + | + |
| Fu 2015 | + | + | + |
| Kane 2010 | ! | + | ! |
| Kane 2011 | + | + | + |
| Kane 2012 | + | + | + |
| Keck 2007 | ! | + | ! |
| Kramer 2007 | + | + | + |
| Liebowitz 2010 | ! | + | ! |
| Macfadden 2009 | + | + | + |
| Marcus 2011 | + | + | + |
| Newcomer 2008 | + | + | + |
| Ozawa 2019 | + | ! | ! |
| Peuskens 2007 | ! | + | ! |
| Pigott 2003 | + | + | + |
| Quiroz 2010 | + | ! | ! |
| Rouillon 2008 | + | + | ! |
| Rui 2014 | + | + | + |
| Stroup 2013 | + | + | + |
| Suppes 2009 | + | + | + |
| Takeuchi 2014 | ! | + | ! |
| Tohen 2004 | + | + | + |
| Tohen 2006 | ! | + | + |
| Tsuboi 2015 | + | + | + |
| Wang 2010 | + | + | + |
| Weiden 2019 | ! | + | ! |
| Weisler 2011 | + | + | + |
| Weiss 2016 | ! | + | ! |
| Yatham 2013 | ! | + | ! |
| Carlson 2012 | ! | + | ! |
| Young 2014 | + | + | + |
| Rapaport 2006 | + | + | + |

| | |
|---|---|
| + | Low risk |
| ! | Some concerns |
| - | High risk |
| D1 | Randomisation process |
| D3 | Missing outcome data |
