# Supplementary figures and images for: Reversibility of Antipsychotic-Induced Weight Gain: A Systematic Review and Meta-Analysis
Source: Front Endocrinol (Lausanne). 2021 Jul 28;12:577919. doi: 10.3389/fendo.2021.577919 (PMC8355990; doi:10.3389/fendo.2021.577919)

Funnel Plot of Standard Error by Difference in means

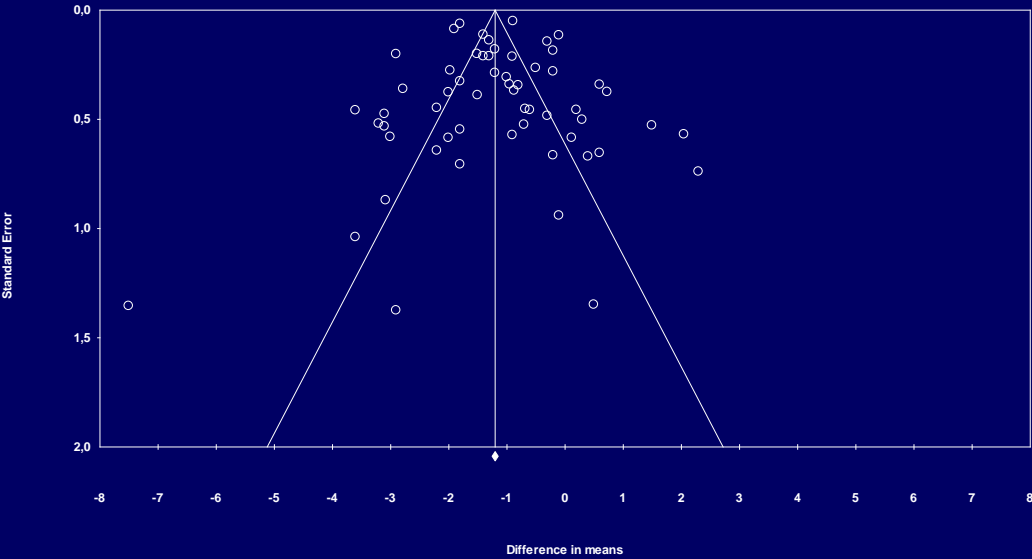

Supplement: Supplementary file 2 [file DataSheet_2.pdf]

Funnel Plot of Standard Error by Difference in means

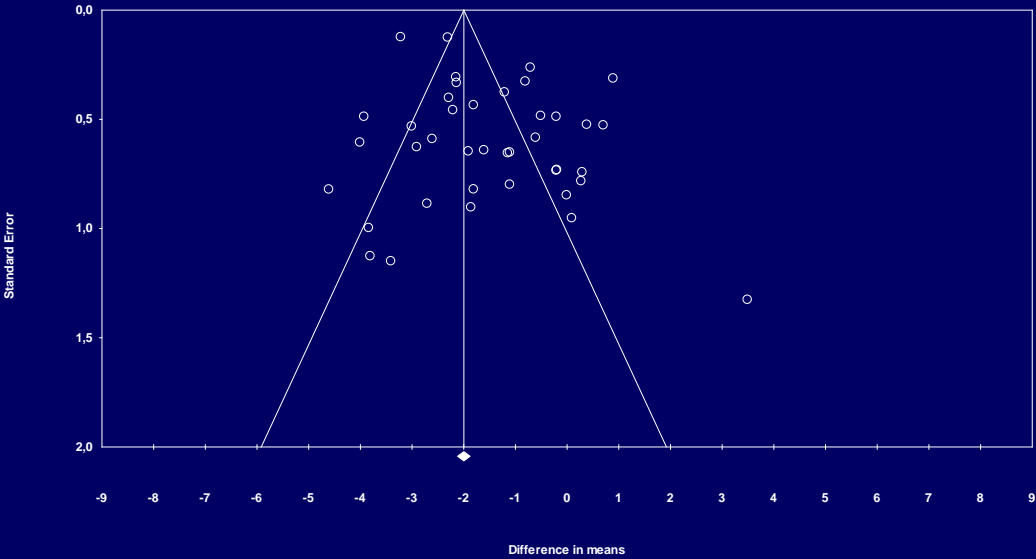

Supplement: Supplementary file 3 [file DataSheet_3.pdf]
